# The Modulation of NMDA and AMPA/Kainate Receptors by Tocotrienol-Rich Fraction and Α-Tocopherol in Glutamate-Induced Injury of Primary Astrocytes

**DOI:** 10.3390/biomedicines5040068

**Published:** 2017-12-01

**Authors:** Zahra Abedi, Huzwah Khaza’ai, Sharmili Vidyadaran, Mohd Sokhini Abd Mutalib

**Affiliations:** 1 Department of Biomedical Science, Faculty of Medicine and Health Science, University Putra Malaysia, Jalan Upm, 43400 Serdang, Malaysia; zahra.ab1983@gmail.com; 2 Department of Pathology, Faculty of Medicine and Health Science, University Putra Malaysia, Jalan Upm, 43400 Serdang, Malaysia; sharmili@upm.edu.my; 3 Department of Nutrition and Dietetics, Faculty of Medicine and Health Science, University Putra Malaysia, Jalan Upm, 43400 Serdang, Malaysia; sukhini@upm.edu.my

**Keywords:** vitamin E, tocotrienol-rich fraction, alpha tocopherol, glutamate toxicity, primary astrocytes

## Abstract

Astrocytes are known as structural and supporting cells in the central nervous system (CNS). Glutamate, as a main excitatory amino acid neurotransmitter in the mammalian central nervous system, can be excitotoxic, playing a key role in many chronic neurodegenerative diseases. The aim of the current study was to elucidate the potential of vitamin E in protecting glutamate-injured primary astrocytes. Hence, primary astrocytes were isolated from mixed glial cells of C57BL/6 mice by applying the EasySep^®^ Mouse CD11b Positive Selection Kit, cultured in Dulbecco’s modified Eagle medium (DMEM) and supplemented with special nutrients. The IC_20_ and IC_50_ values of glutamate, as well as the cell viability of primary astrocytes, were assessed with 100 ng/mL, 200 ng/mL, and 300 ng/mL of tocotrienol-rich fraction (TRF) and alpha-tocopherol (α-TCP), as determined by an 3-(4,5-dimethylthiazol-2-yl)-2,5-diphenyltetrazolium bromide (MTT) assay. The mitochondrial membrane potential (MMP) detected in primary astrocytes was assessed with the same concentrations of TRF and α-TCP. The expression levels of the ionotropic glutamate receptor genes (*Gria2*, *Grin2A*, *GRIK1*) were independently determined using RT-PCR. The purification rate of astrocytes was measured by a flow-cytometer as circa 79.4%. The IC_20_ and IC_50_ values of glutamate were determined as 10 mM and 100 mM, respectively. Exposure to 100 mM of glutamate in primary astrocytes caused the inhibition of cell viability of approximately 64.75% and 61.10% in pre- and post-study, respectively (*p* < 0.05). Both TRF and α-TCP (at the lowest and highest concentrations, respectively) were able to increase the MMP to 88.46% and 93.31% pre-treatment, and 78.43% and 81.22% post-treatment, respectively. Additionally, the findings showed a similar pattern for the expression level of the ionotropic glutamate receptor genes. Increased extracellular calcium concentrations were also observed, indicating that the presence of vitamin E altered the polarization of astrocytes. In conclusion, α-TCP showed better recovery and prophylactic effects as compared to TRF in the pre-treatment of glutamate-injured primary astrocytes.

## 1. Introduction

Neurodegenerative diseases are considered the next great public health challenge and represent a prevalent worldwide health concern [1]. The limitation of available drugs and lack of potent therapeutic agents against neurotoxicity-induced neurological disorders have spurred rapid developments in neuroprotection-related research. Increased concentrations of glutamate in the CNS lead to oxidative stress [2,3,4], which contributes to nerve and glial cell death, subsequently leading to neurodegenerative disorders. According to Coyle and Puttfarcken [5], glutamate toxicity is a kind of brain-cell death, which is associated with many types of chronic neurodegenerative diseases such as Alzheimer’s, Parkinson’s, and Huntington’s diseases.

Glutamate plays a major role as a neurotransmitter in the CNS. Elevated levels of extracellular glutamate, however, are neurotoxic to nerve cells [5,6]. Glutamate is considered a predominant excitatory amino acid neurotransmitter that can be found in the mammalian CNS; it can be toxic. It has also been suggested that it plays a crucial role in the development of neurodegenerative diseases [7]. Glutamate-mediated injury is a main contributor to pathological cell death in the nervous system [8].

There are two types of glutamate-induced toxicity: receptor-initiated excitotoxicity and non-receptor-mediated oxidative toxicity [8]. Receptor-mediated glutamate excitotoxicity involves excessive stimulation of the glutamate receptors (GluRs), which subsequently leads to excessive calcium ion (Ca^2+^) influx and activates the cell death cascade involving the accumulation of mitochondrially-generated reactive oxygen species (ROS).

There are two main subtypes of GluRs: ionotropic and metabotropic. The ionotropic glutamate receptor (iGluR) family can be further categorized according to activation by specific agonists into three subtypes: α-amino-3-hydrox-5-methyl-4-isoxazolepropionate (AMPA), kainite (KA), and *N*-methyl-d-aspartate (NMDA) receptors [9]. The AMPA receptor mediates fast synaptic transmission in the CNS. KA receptors are found mainly in the forebrain [9], but in lower numbers than the AMPA receptors. The KA receptor plays roles in both the pre- and post-synaptic regions. NMDA is important for controlling synaptic plasticity and memory function. Activation of the NMDA receptors is voltage-dependent, leading to the opening of ion channels that are non-selective for cations. The metabotropic glutamate receptor (mGluR) is linked to the G protein, which regulates the intracellular secondary messengers (e.g., inositol phosphate) [10,11]. Eight mGluR subtypes have been identified using molecular biology techniques. The stimulation and opening of iGluRs can initiate Na^+^ or both Na^+^ or Ca^2+^ influx, which subsequently leads to the release of glutamate and finally causes the progressive decrease of neuronal cells associated with many neurological diseases [12]. Studies have shown that excessive extracellular glutamate causes nerve cell death via stimulation of the ionotropic glutamate receptors in the case of stroke and trauma [13].

In the case of a cerebral insult, the role of astrocytes in improving neuronal survival and recovery is increasingly recognized. A large body of recent studies on neuroprotection has focused on improving neuronal survival [14]; however, a main simultaneous effect of ischemic infarction is the death of glia, especially astrocytes. Many studies have shown that astrocytes are damaged after the onset of neurodegenerative disorders [15,16]. For instance, a study by Chen, Liao [17] showed that stimulating astrocytes with glutamate causes cell swelling and cell death. With regard to the significance of astrocyte survival in the occurrence of glutamate insult, it is highly desirable to find a treatment approach to prevent the excitotoxicity that causes cell death. It has been shown that astrocytes treated with vitamin E are able to resist glutamate excito-toxicity [17].

Vitamin E is composed of eight different isoforms: four tocopherols (α-, β-, γ-, δ-) and four tocotrienols (α-, β-, γ-, δ-). The neuroprotective properties of vitamin E have been identified [18]. Tocotrienol is a vitamin E isoform consisting of a chromanol head and a phytyl tail. The structure of tocotrienol is similar to that of tocopherol. However, its side chain or phytyl tail contains a double bond at the 3′, 7′, and 11′ positions. It has higher antioxidant activity than other vitamin E isoforms, i.e., tocopherol [19,20]. Most of the above-mentioned isoforms are focused on neuronal cell protection rather than astrocytes. Tocotrienol-rich fraction (TRF) and alpha-tocopherol (α-TCP) have potent antioxidant, anti-cancer, anti-inflammation, and cholesterol-lowering properties [21,22,23,24,25]. Thus, interest in studying the neuroprotective effect of TRF and α-TCP has increased over time. Hence, the present study attempts to investigate the effects of vitamin E in preventing glutamate excitotoxicity and the method of recovery by vitamin E, in terms of the dose response, viability, and ionotropic glutamate receptor genes in primary astrocytes. The prophylactic and preventive properties of tocopherol in neurodegeneration are expected to be achieved and alternative therapeutics based on nutrition are offered.

## 2. Results

### 2.1. Isolation and Purification of Primary Astrocytes

**Figure 1:**
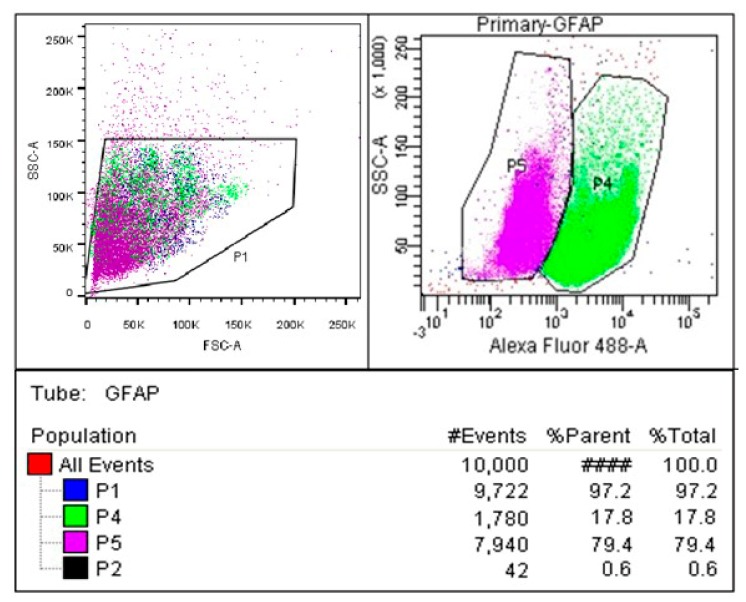
Glial fibrillary acidic protein (GFAP) immunophenotyping to determine the purity of astrocyte culture. Cultures were stained with GFAP antibodies tagged with FITC (Fluorescein Isothiocyanate) and analyzed on a flow cytometer. P1 shows the population of cells; P2 shows the debris cells which are located in the lower left-hand side of scatter plots; P4 shows the primary astrocyte cells; P5 shows the contaminated cells. The plot shows that 79.4% of cells in the culture are GFAP+.

In primary astrocyte isolation, microglia contamination in the culture was eliminated using CD11b for positive selection and l-leucine methyl ester (LME) for negative selection. Primary astrocytes were cultured and maintained in Dulbecco’s modified Eagle medium (DMEM). Primary astrocyte proliferation and differentiation in primary culture were assessed by flow cytometry. Primary astrocytes positive to Glial fibrillary acidic protein (GFAP) isolated by negative selection were 79.40% pure (Figure 1).

### 2.2. Cell Viability of Glutamate-Injured Astrocytes against Vitamin E Treatment

**Figure 2:**
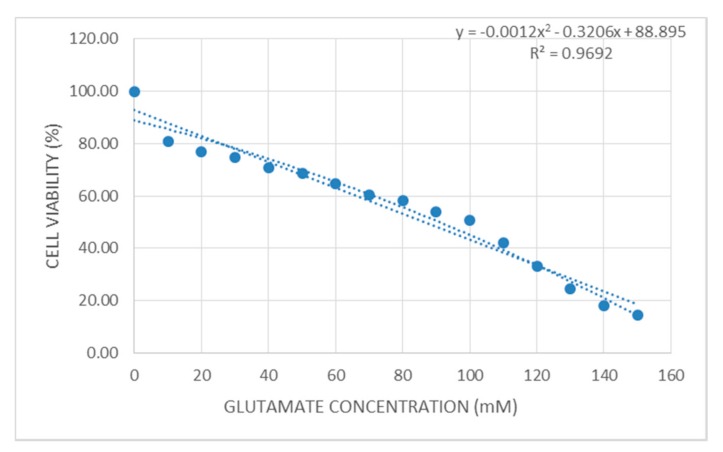
The effect of various concentrations of glutamate on primary astrocyte cells. Cultures were treated with various concentrations of glutamate for 24 h, y is showing the cell viability, x is showing the glutamate concentration and R^2^ is showing coefficient of determination. IC_20_ and IC_50_ were calculated based on an equation with almost 97% accuracy. The figure shows 80% and 50% cell viability in 10 mM and 100 mM glutamate concentrations, respectively.

**Figure 3:**
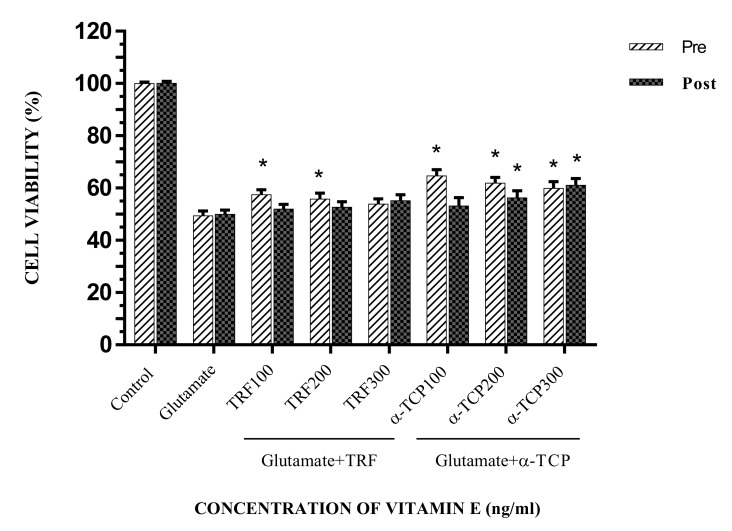
The effect of pre- and post-treatment with TRF and α-TCP against 100 mM glutamate on cell viability in astrocytes. Data represent the mean ± SEM of three independent experiments (*n* = 3 in each experiment). * *p* < 0.05, TRF- and α-TCP-treated groups compared with the glutamate-treated group. This shows that TRF and α-TCP, at a low concentration (100 ng/mL) in pre-incubation, and at a high concentration (300 ng/mL) in post-incubation, exerted potential prophylactic effects against glutamate toxicity in the primary astrocyte.

In the dose-response study, the primary astrocytes were exposed to various concentrations of glutamate ranging from 10 mM to 150 mM glutamate and the cell viability was determined after 24 h of incubation. Cell viability was reduced as the concentrations of glutamate increased from 10 mM to 150 mM. The IC_20_ and IC_50_ values of glutamate were determined by the 3-(4,5-dimethylthiazol-2-yl)-2,5-diphenyltetrazolium bromide (MTT) assay and analyzed by Polo plus software (Probit analysis) at 10 mM and 100 mM, respectively (Figure 2). The effects of TRF and α-TCP upon glutamate-induced cytotoxicity were evaluated via the MTT cell viability assay. Exposure to 100 mM of glutamate in primary astrocytes caused an inhibition of cell viability of approximately 50%. Pre-treatment with 100 ng/mL, 200 ng/mL, and 300 ng/mL of TRF and α-TCP increased the viability of the cell significantly, with averages of 57.59%, 55.89%, and 53.94%, and 64.75%, 61.98%, and 59.94%, respectively. A short pre-incubation time (5 min) was utilized to compare the efficiency between TRF and α-TCP uptake. This shows that TRF and α-TCP, at a low concentration and over a short pre-incubation period, exerted potential prophylactic effects against glutamate toxicity in the primary astrocyte. The proliferation rates for 100 ng/mL, 200 ng/mL, and 300 ng/mL of TRF-treated cells were 52.05%, 52.77%, and 55.29%, respectively, and this was observed in post-treatment as TRF and α-TCP were given after 30 min of incubation with glutamate. Meanwhile, α-TCP-treated cells exhibited cell survival rates of 53.27%, 56.31%, and 61.10% after the glutamate challenge (Figure 3).

### 2.3. Effects of Vitamin E in Preserving Mitochondrial Membrane Potential of Primary Astrocytes after Glutamate Excitotoxicity

**Figure 4:**
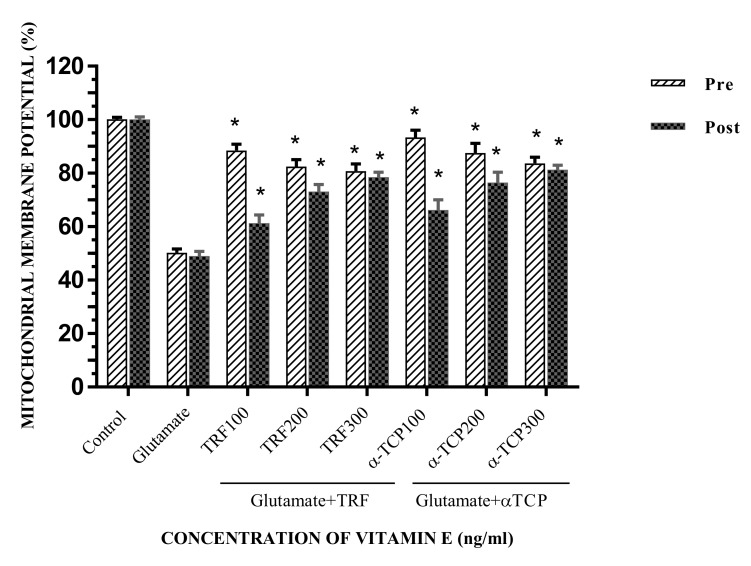
The effect of pre- and post-treatment with TRF and α-TCP on glutamate-injured astrocytes with respect to mitochondrial membrane potentiality. Data represent the mean ± SEM of three independent experiments (*n* = 3 in each experiment). * *p* < 0.05, TRF- and α-TCP-treated groups compared with the glutamate-treated group. This shows that TRF and α-TCP, at a low concentration (100 ng) in pre-incubation, and at a high concentration (300 ng) in post-incubation, demonstrated better prophylactic properties against the toxicity of glutamate in primary astrocytes.

A pre-treatment study of the mitochondrial membrane potential (MMP) assay clarified the effects of vitamin E at 100 ng/mL, 200 ng/mL, and 300 ng/mL. Figure 4 illustrates the results of pre-treatment of this assay with TRF and α-TCP, which indicates that MMP reached 88.46%, 82.42%, and 80.74%, and 93.31%, 87.51%, and 83.70%, respectively. TRF and α-TCP, at low concentrations and after 24 h of pre-incubation, demonstrated better prophylactic properties against the toxicity of glutamate in primary astrocytes. On the other hand, 100 ng/mL of TRF and α-TCP gave the highest MMP values.

Next, a post-treatment MMP assay was selected to determine the recovery effects of vitamin E upon glutamate insult in primary astrocytes. Figure 4 demonstrates that both TRF and α-TCP increased MMP upon glutamate challenge. The MMP increased to 61.21%, 73.01%, and 78.43% with treatment with TRF at 100 ng/mL, 200 ng/mL, and 300 ng/mL, respectively. α-TCP treatment illustrated MMP values of 66.21%, 76.46%, and 81.22% at 100 ng/mL, 200 ng/mL, and 300 ng/mL, respectively. Post-treatment with both TRF and α-TCP at high concentrations (300 ng/mL) was able to prevent a decrease in the level of MMP for glutamate-injured primary astrocytes.

### 2.4. Effect of Vitamin E on the Expression of Ionotropic Glutamate Receptors Genes

Gene expression analysis was determined by computing the Cq values acquired utilizing the method by Fleige, Walf [26], and Pfaffl [27]; the results are represented as the fold ratio, which was further converted to log 10 values to simplify data presentation. In this study, the gene expression ratio was calculated based on the amplification efficiency of the target gene with the Cq value of each treated sample versus the control (untreated cells) in comparison to the *ACTB* gene. The earlier findings recognized that TRF and α-TCP protected and aided in the recovery of primary astrocyte cells from glutamate toxicity. Further experiments were completed to determine the level of cell injury caused by glutamate and to examine the effects of vitamin E in recuperating the cell damage. In this study, the level of cell injury was evaluated through *Gria2*, *GRIK1*, and *Grin2A* expression, known as ionotropic glutamate receptor genes; *ACTB* was utilized as the internal control. These results show that *Gria2*, *Grik1*, and *Grin2a* are significantly expressed in the glutamate-induced injury of primary astrocyte cells compared to the negative control. Pre- and post-treatment with 100–300 ng/mL TRF and α-TCP significantly reduced the transcription levels of *Gria2*, *GRIK1*, and *Grin2A* in comparison to the positive control, respectively. Our findings provide evidence that mRNA regulation of glutamate receptor levels in astrocytes is required to prevent excitotoxic death. Glutamate-injured cells were rescued upon pre- and post-treatment with TRF and α-TCP, as these prevented the depolarization of astrocytes, suggesting that excess glutamate signaling was the cause of astrocyte cell death. Both the cell loss and concomitant climbing defects in the glutamate-injured cells were rescued by lowering the glutamate receptor levels in these cells. This suggests that they may block voltage-dependent Mg^2+^ in the NMDA receptor subunit (NMDARs), and exhibit higher Ca^2+^ permeability in glutamate-injured astrocytes [28,29].

**Figure 5:**
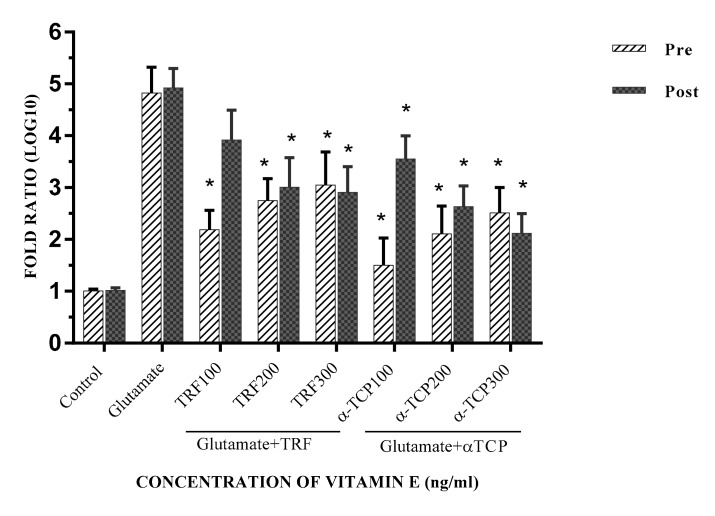
*Gria2* mRNA expression in primary astrocytes with TRF and α-TCP pre-treatment against glutamate insult. Fold change of *Gria2* normalized to *ACTB* mRNA expression. Data are the mean ± SEM of three independent experiments (*n* = 3 in each experiment). * *p* < 0.05, TRF- and α-TCP-treated groups compared with the glutamate-treated group. Pre- and post-treatment with 100–300 ng/mL TRF and α-TCP significantly reduced the transcription levels of *Gria2* in comparison to the positive control (glutamate).

**Figure 6:**
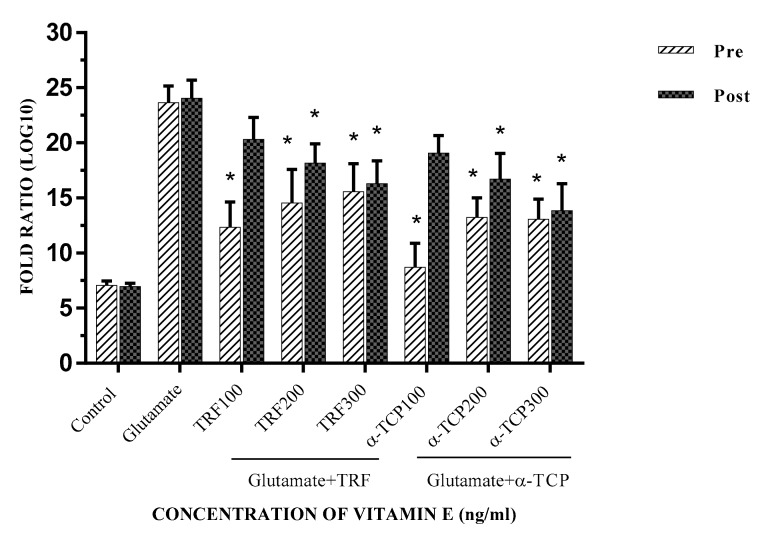
*GRIK1* mRNA expression in primary astrocytes with TRF and α-TCP pre-treatment against glutamate insult. Fold change of *GRIK1* normalized to *ACTB* mRNA expression. Data are the mean ± SEM of three independent experiments (*n* = 3 in each experiment). * *p* < 0.05, TRF- and α-TCP-treated groups compared with the glutamate-treated group. Pre- and post-treatment with 100–300 ng/mL TRF and α-TCP significantly reduced the transcription levels of *GRIK1* in comparison to the positive control (glutamate).

**Figure 7:**
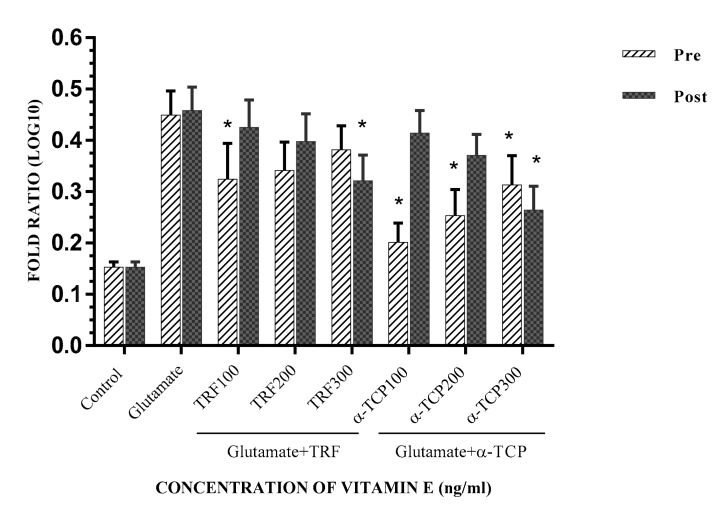
*Grin2A* mRNA expression in primary astrocytes with TRF and α-TCP pre-treatment against glutamate insult. Fold change of *Grin2A* normalized to *ACTB* mRNA expression. Data are the mean ± SEM of three independent experiments (*n* = 3 in each experiment). * *p* < 0.05, TRF- and α-TCP-treated groups compared with the glutamate-treated group. Pre- and post-treatment with 100–300 ng/mL TRF and α-TCP significantly reduced the transcription levels of *Grin2A* in comparison to the positive control (glutamate).

The mRNA expression for *Gria2* was most significantly decreased in the pre-treatment with 100 ng/mL of α-TCP, whereas the effects of TRF (*p* < 0.001 and *p* < 0.01) were significantly reduced as compared to post-treatment. However, the effect of α-TCP in the pre- and post-treatment study was more significant (Figure 5). Pre- and post-supplementation primary astrocyte cells with 100–300 ng/mL α-TCP/TRF also resulted in a moderately reduced expression of the *Grik1* gene (Figure 6). Although the expression of *Grin2a* was slightly decreased in the pre- and post- α-TCP and TRF treatment groups, the changes were still significant (*p* < 0.04). (Figure 7). For both *Grik1* and *Grin2a*, as well as *Gri2a*, the lowest dose (100 ng/mL) and the highest dose (300 ng/mL) of α-TCP and TRF in pre- and post-treatment, respectively, showed the most significant suppression effects with respect to the control of mRNA expression in glutamate-injured primary astrocytes.

### 2.5. Calcium (Ca^2+^) Influx in Primary Astrocytes upon Glutamate Toxicity

**Figure 8:**
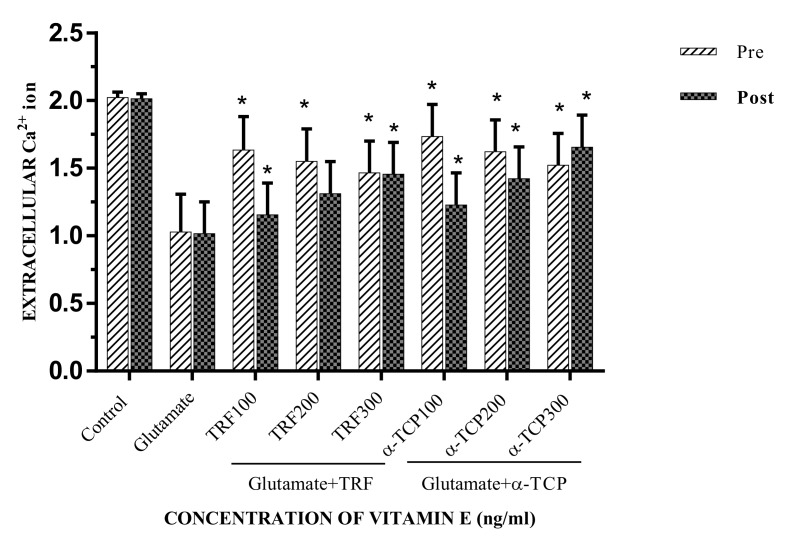
Pre- and post-treatment of TRF and α-TCP against glutamate-injured astrocytes in extracellular Ca^2+^ ions. Data represent the mean ± SEM of three independent experiments (*n* = 3 in each experiment). * *p* < 0.05, TRF- and α-TCP-treated groups compared with the glutamate-treated group. This shows that TRF and α-TCP, at a low concentration (100 ng) in pre-incubation, increased the levels of extracellular Ca^2+^ ions, while a high concentration of TRF and α-TCP (300 ng) in post-incubation decreased the level of extracellular Ca^2+^ ions against the toxicity of glutamate in primary astrocytes.

The levels of extracellular Ca^2+^ ions were examined using a colorimetric calcium assay. The primary astrocytes were exposed to 100 mM glutamate for 24 h. The extracellular Ca^2+^ ion value was 1.02 ± 0.03 mg/mL, which was significantly decreased compared to the negative control (*p* < 0.05) (Figure 8).

The present data shows that the extracellular Ca^2+^ ion levels were significantly increased in the primary astrocytes, which were treated with 100 to 300 ng/mL of TRF and α-TCP in both the pre- and post-treatment study groups, while doses of 100 ng/mL and 300 ng/mL of α-TCP reveled the greatest significant difference in the pre- and post-treatment groups, respectively.

## 3. Discussion

There are various protocols available for the isolation of astrocytes and microglia from rodent brain tissue. These reports are similar in the basic principle of their methods. However, discrepancies were observed in the protocol. These included magnetic separation of microglia and astrocytes from mixed glia culture using columns [30,31], magnetic separation without columns [32], brief shaking [33,34], and the elimination of microglia from confluent monolayers of primary astrocytes using LME [35]. In our research, the yield and purity of astrocytes were consistent in magnetic sorting (EasySep^®^ Mouse CD11b Positive Selection Kit) and in the use of LME in negative selection. The astrocytes remaining in the mixed cultures were obtained at 79.4% purity by treatment with LME and a subsequent vigorous shaking step to eliminate any remaining microglia and neurons. Furthermore, no cross-contamination was detected in the glial cultures. This technique provides a simple and reliable method for achieving highly purified preparations of astrocytes from primary mixed glia culture for the study of brain cell biology and neurodegenerative diseases.

Through the dose-response curve (Figure 2), the half maximal inhibitory concentration (IC_50_) of glutamate for primary astrocyte cells was determined (the concentration that inhibits 50% of the cell population). It was expected that the cell viability would drop significantly on exposure to high concentrations of glutamate. The current observation illustrates that 100 mM of glutamate is required to cause the death of 50% of primary astrocytes. This observation is similar to that of a study on SH-SY5Y neuronal cells (a subclone of SK-N-SH neuronal cells), against glutamate challenge for hours [36]. Previous observation showed that the exposure of CRL-2020 astrocyte cells to 180 mM glutamate reduced cell viability by more than 50% in 24 h [3]. This shows that primary astrocytes do not have the capability to survive in an environment with a higher concentration of glutamate. The observation proposes an excessive level of glutamate-induced cellular impairment in primary astrocytes.

Primary astrocyte cells were supplemented with 100–300 ng/mL of TRF and α-TCP before and after glutamate injury. The MTT assay revealed that 100 ng/mL TRF and α-TCP pre-treatment resulted in the highest cell viability. In the post-treatment study, cell viability was highest following treatment with 300 ng/mL TRF and α-TCP. Pre-treatment also increased cell viability significantly compared to the positive control (*p* < 0.05). This suggests that micromolar concentrations of TRF and α-TCP are sufficient for protection against neurodegeneration. This is similar to the findings in a previous study [2,3,37]. Supplementing the cells with 100–300 ng/mL TRF/α-TCP increased cell viability after 24 h of incubation. A study by Osakada [38] showed that, at a concentration of 1–10 μM, α-TCP protects striatal neurons against cytotoxicity induced by l-buthionine-*S*,*R*-sulfoximine (BSO) via the reduction of oxidative stress. This isomer also has the highest bioavailability of α-TCP, compared to the other isomers, due to recognition by the α-TCP transfer protein (α-TTP) [39]. The results thus suggest that vitamin E is effective in aiding astrocyte cell survival following glutamate toxicity.

TRF/α-TCP pre- and post-treatment significantly increased the MMP, demonstrating a protective effect on the mitochondrial membrane compared to the positive control (*p* < 0.05). In accordance with the findings of a previous study which investigated patients with abetalipoproteinemia, chronic disorders of fat absorption, and ataxia, as well as comparative neuropathological studies in humans and experimental animals [40], vitamin E played a significant role in maintaining regular neurological structure and function. We also observed that α-TCP had better neuroprotective and recovery effects compared to TRF. It was previously noted that α-TCP had compelling effects against glutamate cytotoxicity [3,41]. Also, α-TCP was effective for inhibiting oxidative injury in rat mitochondria [42]. In addition, nanomolar concentrations of α-TCP protected against glutamate-induced neuronal death by suppressing inducible pp60 c-Src kinase activation Sen, Khanna [43]. The activation of c-Src leads to promotion of the survival, angiogenesis, proliferation, and invasion pathways related to cancer formation. The previous results also show that α-TCP has a greater antioxidant value, where it is effective in inhibiting chronic diseases related to oxidative stress, such as malignancy, coronary heart diseases, and stroke [44]. The key role of α-TCP is to quench the chain reaction of lipid peroxidation to protect the cell membrane and low-density lipoprotein (LDL) from oxidative collapse [45]. α-TCP can involve cell signaling activities such as the modulation of phospholipase A_2_ activity, regulation of protein kinase C, and inhibition of cyclooxygenase-2 activity.

Recent available data suggest that glial iGluRs respond to glutamate that is freed from synaptic terminals or stimulated axons. It was also evident that α-TCP and TRF supplementation significantly suppressed *Gria2*, *GRIK1*, and *Grin2A* expression, respectively, upon glutamate challenge. The results of this study combined with previous work suggest that the *Gria2* gene, which encodes the GluR2 subunits of the AMPA receptor, is more abundantly expressed in the glutamate-induced injury of primary astrocyte cells than the *Grik1* and *Grin2a* genes [46,47]. Compared to the other receptors, evidence shows the more influential role of the AMPA receptor in glial glutamate excitotoxicity [48,49]. We also observed that α-TCP increased the all-viability of astrocytes via a decrease in the iGluRs, perhaps due to the reduced polarity of the cells. It was previously mentioned that α-TCP has enforcing effects against glutamate cytotoxicity.

Araque, Parpura [50] believe that glial AMPA receptors have a reasonable Ca^2+^ permeability and an increase in astroglial Ca^2+^ ions will lead to a release of glutamate and subsequent neuronal stimulation. The data attained in the present study substantiate the same result.

Moreover, high concentrations of extracellular glutamate generated, for instance, after traumatic and ischemic CNS injury, will result in the overstimulation of AMPA, kainite, and NMDA receptors, respectively, and, as a consequence, also increase the influx of Na^+^ and Ca^2+^ ions through the pores formed by these receptors [51]. The present study proves that glutamate excitotoxicity in glial cells is exclusively mediated by AMPA and kainate receptors [52]. The result of this study postulates that vitamin E treatment (α-TCP and TRF) reduces oxidative stress via mitochondrial membrane protection and the prevention of AMPA/Kainate and NMDA receptor activity, as well as permeability to the Ca^2+^ ion.

The results show that TRF and α-TCP can reduce the Ca^2+^ permeability in primary astrocytes by down regulating the iGluR genes. This finding demonstrates that TRF and α-TCP were able to reduce intracellular Ca^2+^ and Ca^2+^ influx during glutamate toxicity. In other words, extracellular Ca^2+^ increased when the cells were treated with 100–300 ng/mL TRF and α-TCP in comparison to the positive control (Figure 8). Hence, in the pre-treatment study, both TRF and α-TCP showed a considerably significant effect in reducing Ca^2+^ influx at 100 ng/mL. However, in the post-treatment study, both TRF and α-TCP had an effect on Ca^2+^ influx at 300 ng/mL in the astrocyte primary culture. Accordingly, synchronous with the decline of the Ca^2+^ influx, intracellular Na^+^ ions decreased with a rise in intracellular K^+^ concentration [53].

Ionotropic glutamate receptors mediating excitotoxicity are permeable to Ca^2+^, a property that varies from one receptor subclass to another. High concentrations of extracellular glutamate are generated, for instance, after traumatic and ischemic CNS injury, resulting in the overstimulation of AMPA, kainite, and NMDA receptors and, as a consequence, an increase in the influx of Na^+^ and Ca^2+^ ions through the pores formed by these receptors [51]. This intracellular Ca^2+^ ion acts as an important second messenger, activating several intracellular signaling cascades. For example, some Ca^2+^ ions bind to calmodulin, and this complex in turn activates several protein kinases, including calcium/calmodulin-dependent protein kinase, or CAM kinase, which affects the AMPA receptors. CAM kinase phosphorylates AMPA receptors and increases their conductance to Na^+^. CAM kinase also promotes the movement of AMPA receptors from the intracellular store into the membrane, making more receptors available for stimulation [54]. Due to the high antioxidant effects of α-TCP involving cell signaling activities and regulating protein kinases, perhaps α-TCP will be able to inhibit the CAM kinase and reduce AMPA receptor activity and NMDA channel, thereby decreasing their permeability to Ca^2+^ and Na^+^ ions. Additionally, the collapse of MMP will be inhibited by α-TCP, and the secondary increase in cytosolic Ca^2+^ concentration and release of cytochrome C can be reduced.

Na+ influx can in turn trigger a secondary increase in the intracellular Ca^2+^ concentration through the activation of voltage-gated Ca^2+^ channels and reverse operation of the Na^+^/Ca^2+^ exchange. The present data also confirm that glutamate-excitotoxicity in glial cells is exclusively mediated by AMPA and kainate receptors [52].

## 4. Material and Methods

Utilization and care of animals (C57BL/6 mice pups) were approved by the Animal Care and Use Committee (ACUC, Approvl ID UPM/FPSK/PADS/BRUUH/00163, 12 September 2015.), Faculty of Medicine and Health Sciences, Universiti Putra Malaysia. Fetal bovine serum (FBS), penicillin/streptomycin, trypsin, phosphate-buffered saline (PBS), Dulbecco’s modified Eagle medium (DMEM), and additional nutrition were acquired from Invitrogen (Carlsbad, CA, USA). Polystyrene round-bottom tubes were acquired from BD Biosciences (San Jose, CA, USA). EasySep^®^ Mouse CD11b Positive Selection Kits were obtained from STEMCELL Technologies (Vancouver, BC, Canada); fluorescently-conjugated antibodies (Glial fibrillary acidic protein (GFAP), FITC 490–525 nm AlexafluorR 488, Dylight, Thermo Fisher, Waltham, MA, USA) were obtained from Abnova (Taipei, Taiwan); and propidium iodide (PI) as a stain buffer was obtained from Sigma-Aldrich (St. Louis, MO, USA). 3-(4,5-dimethylthiazol-2-yl)-2,5-diphenyltetrazolium bromide (MTT) powder was acquired from Phytotechnology Laboratories (Overland Park, KS, USA). Dimethyl sulfoxide (DMSO) and Rhodamine 123 were attained from Sigma (St. Louis, MO, USA). We obtained the DNase I RNase-free Kit, the GeneJET RNA Purification Kit, and the DreamTaq Green PCR Master Mix from Thermo Scientific (Waltham, MA, USA). The RevertAid First Strand cDNA Synthesis Kit and SsoFast EvaGreen Supermix were obtained from Bio-Rad (Hercules, CA, USA).

### 4.1. Preparation of Mixed Glia Cultures

Primary mixed glia cultures were extracted from the brain of postnatal C57BL/6. Pups between postnatal days one and five were subjected to a carbon dioxide overdose; brains were examined and kept in ice-cold DMEM. Meninges and vessels were thoroughly expelled and the tissue was minced with a sterile surgical tool. A total of 10 mL of assimilation blend (1 mL of 0.25% trypsin-ethylenediaminetetraacetic (trypsin-EDTA), 10 μL of 10 mg/mL DNase, and 9 mL DMEM) were added to the minced tissue (from three brains) and the blend was triturated against the base of the culture dish to separate cell bunches. The mixture was transferred to a 50 mL tube and raised up in a shaking hatchery at 37 °C at 4× *g* for 10 min. The tube was left for 10 min to permit the bunches to reside. The supernatant was collected with a serological pipette and sifted through a 70 μm cell strainer. A similar volume of supplementary media (DMEM, 15% FBS) was put through the strainer and the cells were pelleted at 448× *g* for 10 min. The supernatant was disposed of and the pellet was resuspended in the entire media and seeded into poly-l-lysine-covered carafes (1 brain: three 25 cm^2^ cups). Poly-l-lysine coating was prepared a day before the cell isolation by layering the flask with 0.1 mg/mL poly-l-lysine for 10 min at room temperature, followed by washing with 1× PBS and drying overnight at 37 °C. Cells were left in the incubator for around 15–20 days until they were confluent.

### 4.2. Isolation of Microglia from Mixed Glia Cultures by Magnetic Sorting

Magnetic sorting of microglia from mixed glial was accomplished utilizing the EasySep^®^ Mouse CD11b Positive Selection Kit. Briefly, confluent mixed glia cultures were gained utilizing 0.25% trypsin-EDTA, levigated to separate the cell clumps, and put through a 70 μm cell strainer to get a single cell suspension. Cells were pelleted and suspended in 1 mL of DMEM to get a final concentration of no more than 1.0 × 10^8^ cells/mL. The cell suspension was then relocated into FalconTM 5 mL polystyrene round-bottom tubes and 50 μL of CD11b PE (Purified anti-phycoerythrin) labeling reagent was added. Following incubation at room temperature (RT) for 15 min in the dark, 70 μL of EasySep^®^ PE Selection Cocktail was added and left undisturbed for 15 min on ice. Magnetic nanoparticles were mixed well utilizing a pipette and then 50 μL was added to the cell suspension and incubated on ice for 10 min. Next, the cell suspension was made up to 2.5 mL with PBS, mixed gently using a pipette, and the tube was placed in the EasySep^®^ magnet and set aside for 5 min at RT. The CD11b cells in the cell suspension were poured in a continuous gesture allowing the magnetically labelled cells to be detained by the magnetic field of the EasySep^®^ magnet. The tube was deleted from the magnet and the cells were mixed in 2.5 mL of PBS by gentle pipetting. The tube was put back in the EasySep^®^ magnet and set separately for 5 min. CD11b cells were exhausted as described earlier. The sorting steps were repeated with the initial positive fractions to improve the yield. The retained negative fractions were collected and suspended in DMEM complete medium for the purification of primary astrocytes, and downstream experiments.

### 4.3. Purification of Astrocytes from Mixed Glia Culture

To gain the astrocytes, the flasks from negative selection were treated with 60 mM l-leucine methyl ester (LME, Sigma-Aldrich, St. Louis, MO, USA) for 90 min in 8% CO_2_ at 37 °C [35]. Then, the sides of the flasks were tapped several times to eliminate dead cells. The flasks were washed thrice with DMEM comprising 15% FBS with added antibiotics (100 U/mL penicillin G). Then, cultures were stored in 8% CO_2_ at 37 °C. The typical yield of astrocytes post-treatment with LME was approximately 2 × 10^6^ cells/75 cm^2^ culture flask. Cultures of primary astrocytes were maintained in DMEM with 15% FBS and antibiotics and a change of medium was performed three times.

### 4.4. Determination of Primary Astrocytes Purity by Flow Cytometry

The percentage of primary astrocytes after purification was estimated via flow cytometry. Briefly, in 200 mL of blocking buffer (0.2% bovine serum albumin (BSA) in PBS), 5 × 10^5^ cells were suspended and stained with GFAP antibody (FITC 490–525 nm AlexafluorR 488, Dylight), and then PI was used as a stain buffer for 30 min at room temperature. The cells were subsequently washed with PBS and suspended in 300 mL of blocking buffer. Flow cytometry examination was performed on the BD LSRFortessaTM flow cytometer (BD Biosciences, San Jose, CA, USA) equipped with BD FACSDivaTM software (BD Biosciences).

### 4.5. MTT Assay

The cell viability was assessed via an MTT assay upon treatment with glutamate for 24 h followed by vitamin E supplementation. A study by Sen [55] illustrated that a low concentration of TRF suppresses the glutamate-induced activation of c-Src kinase (Proto-oncogene tyrosine-protein kinase Src). Selvaraju et al. [2,3] demonstrated that concentrations of both TRF and α-TCP at 100 ng/mL, 200 ng/mL, and 300 ng/mL are sufficient for preventing the astrocytes from glutamate-induced cell deaths. In evaluating the prophylactic properties of vitamin E, the cells were pre-treated with 100 ng/mL, 200 ng/mL, and 300 ng/mL of TRF and α-TCP before glutamate injury. The post-treatment study was carried out through supplementing the cell with TRF and a-TCP after the astrocytes had been challenged with glutamate (Table 1). The procedure of the MTT assay was performed by adding 50 µL of MTT to each well, followed by incubation for 4 h. Then, the contents of the well were discarded with a syringe before 100 µL of DMSO was added to each well. The data collected for absorbance, which revealed the viability of the cells, were taken at 570 nm with a background value of 630 nm using the microplate reader. Eventually, the graph of the viability of the cells against the concentration of vitamin E was plotted.

### 4.6. Mitochondria Membrane Potential Assay (MMP Assay)

Upon glutamate challenged and vitamin E intervention, the cells were incubated for 24 h, and then the MMP assay was performed. On the third day (after seeding and treatment), the cells were washed with PBS and stained with 50 µL of Rhodamine 123 for 30 min. Rhodamine 123 is known as a fluorescent detection dye; it binds to the mitochondria of cells and inhibits the electron transport chain (ETC). Thus, in healthy mitochondria, in order to stop the process of ETC and provide high-density fluorescent detection, more Rhodamine 123 is needed. After 30 min of incubation with dye, at a wavelength of 485 nm and emission of 530 nm, the cells were washed with PBS and were read through a fluorescent microplate reader. Consequently, the graph of mitochondrial membrane potentiality against vitamin E was plotted.

### 4.7. End-Point RT-PCR

Primary astrocyte cells, which are nontoxic with a 20% inhibitory concentration (IC_20_) of the glutamate, were employed in RNA extraction to induce minimal injury and to obtain a higher yield of RNA. RNA from cells subjected to glutamate insult and supplementation with vitamin E were extracted and converted to cDNA with the DNase I RNase-free Kit. The cDNA prototype for each sample was standardized to achieve the final concentration to be used in the RT-PCR. The genes of interest containing *GRIK1* (kainate receptor, GluR5) [56,57], *Gria2* (AMPA receptor, GluR2 subunit) [57,58], and *Grin2A* (NMDA receptor, NR2 subunit) [57,59], together with their housekeeping gene, ACTB (Actin, Beta, *homo sapiens*), were utilized in this determination. The original primers were synthesized by FIRST BASE laboratories (Malaysia). Finally, all primers were optimized by IDT (Integrated DNA Technologies, Coralville, IA, USA). Refer to Table 2 for the list of primers used in this study.

### 4.8. RT-PCR

SYBR Green dye was used in the RT-PCR. The intensity of the fluorescence emissions increases as the dye binds to double-stranded DNA. The optimum annealing temperatures (50–60 °C or 55–65 °C for 30 s) were used in the RT-PCR. A master mix for RT-PCR qualified quantification of both the target and reference genes was prepared in a total volume of 20 μL. Reaction mixtures with non-prototype or without cDNA prototype control were incorporated to measure for contamination and nonspecific amplification. In every independent experiment, reference and target cDNA were derived from similar extracted RNA and run simultaneously in the RT-PCR. Three independent experiments, each in triplicate, were accomplished, and the quantitative data gained were averaged based on quantification cycle (Cq) values, which are used for computing the fold expression ratio. *ACTB* was incorporated as the reference gene for normalization of the target genes and for compensating for inter-PCR variation between each RT-PCR experiment. RT-PCR was performed in a MiniOpticon™ Real-Time Detection System (Bio-Rad) with the following cycling conditions: enzyme activation (95 °C for 30 s), 40 cycles of denaturation (95 °C for 5 s), annealing or extension (58.3 °C for 5 s), and melt curve analysis at 65–95 °C in 0.5 °C increments for 5 s.

### 4.9. Extracellular Ca^2+^ Ion Measuring

The levels of extracellular Ca^2+^ ions were determined using a colorimetric calcium assay kit (Abcom, Cambridge, MA, USA). In brief, the cells were exposed to 100 mM glutamate and 100 ng/mL to 300 ng/mL TRF and α-TCP in pre- and post-treatment studies for 24 h. In the following steps, 50 μL of media was added to 90 μL of chromogenic reagent in 96-well plates and mixed gently. In the last step, 60 μL of calcium assay buffer was added in the incubator for 10 min. Samples were obtained with an enzyme-linked immunosorbent assay (ELISA) reader (BioTek ELx800, Winooski, VT, USA) at a 575 nm wavelength.

### 4.10. Data Analysis

All the retrieved data are given as mean ± SEM (standard error of the mean). As for statistical analysis, two-way analysis of variance (ANOVA) was utilized and Tukey’s test was carried out for comparison between each treatment concentration using SPSS statistical analysis (Version 22.0.0.0, IBM SPSS Statistic, Armonk, NY, USA). A *p*-value less than 0.05 was considered statistically significant.

## 5. Conclusions

In conclusion, a significant issue worldwide is the development of a perfect therapy/compound to protect individuals suffering from neurodegenerative disorders. In recent years, scientists have found that astrocytes play a very significant role, rather than just providing support to neurons. In conclusion, vitamin E (α-TCP and TRF), as a chain-breaking antioxidant, has protective and healing properties against glutamate toxicity in primary astrocytes.

The neuroprotective effects of vitamin E might be correlated to antioxidant activity, as both TRF and α-TCP secure and mend neuronal and astrocyte cell injury. We postulate that vitamin E has anti-apoptotic or pro-survival properties in preventing apoptosis or repairing stress-mediated damage in primary astrocytes following glutamate toxicity. Obviously, the action of vitamin E in protecting astrocytes from oxidative stress-mediated cell injury may increase the survivability of the cells, hence reducing the occurrence of Alzheimer’s and other neurodegenerative diseases. Therefore, taking into account the prophylactic role of vitamin E, a consideration of dosage in nutrition-based therapy is important for minimizing neurodegeneration. Clearly, palm TRF and α-TCP have vast potential for development with respect to neurodegenerative diseases.

## Figures and Tables

**Table 1:** Experimental design of vitamin E treatments against glutamate injury.

Sample	Treatment
1	Negative control/untreated sample (cells with medium and absolute ethanol only)
2	Positive control (astrocyte cell, 100 mM of glutamate/astrocytes)
3	100 ng/mL TRF + glutamate
4	200 ng/mL TRF + glutamate
5	300 ng/mL TRF + glutamate
6	100 ng/mL α-TCP + glutamate
7	200 ng/mL α-TCP + glutamate
8	300 ng/mL α-TCP + glutamate

**Table 2:** Summary of primers.

Target Gene	Forward Primer (5′-3′)	Reverse Primer (5′-3′)
*Gria2*	5′-TAGACTCTGGCTCCACTAAAGA-3′	5′-GTAGTCCTCACAAACACAGAGG-3′
*GRIK1*	5′-GTCAGTGCTGTGCAGTCTATT-3′	5′-AGCTGCATAATCTGGGTAAAGG-3′
*Grin2A*	5′-CACGGTCATGCTGAAGAT-3′	5′-TCTTGACGAAGCTGATGAA-3′
*ACTB*	5′-GCTCCGGCATGTGCAARG-3′	5′-CATCACACCCTGGTGCCT-3′
